# AC/DC Magnetic Field Sensing Based on a Piezoelectric Polymer and a Fully Printed Planar Spiral Coil

**DOI:** 10.1021/acsami.4c09409

**Published:** 2024-08-26

**Authors:** Josu Fernández Maestu, Nelson Pereira, Senentxu Lanceros-Méndez

**Affiliations:** † BCMaterials, Basque Center for Materials, Applications and Nanostructures, UPV/EHU Science Park, Leioa 48940, Spain; ‡ Physics Center of Minho and Porto Universities (CF-UM-UP) and LaPMET—Laboratory of Physics for Materials and Emergent Technologies, University of Minho, Braga 4710-057, Portugal; § IKERBASQUE, Basque Foundation for Science, Bilbao 48009, Spain

**Keywords:** piezoinductive effect, magnetoelectrics, PVDF, printed electronics, filler free, magnetic sensors

## Abstract

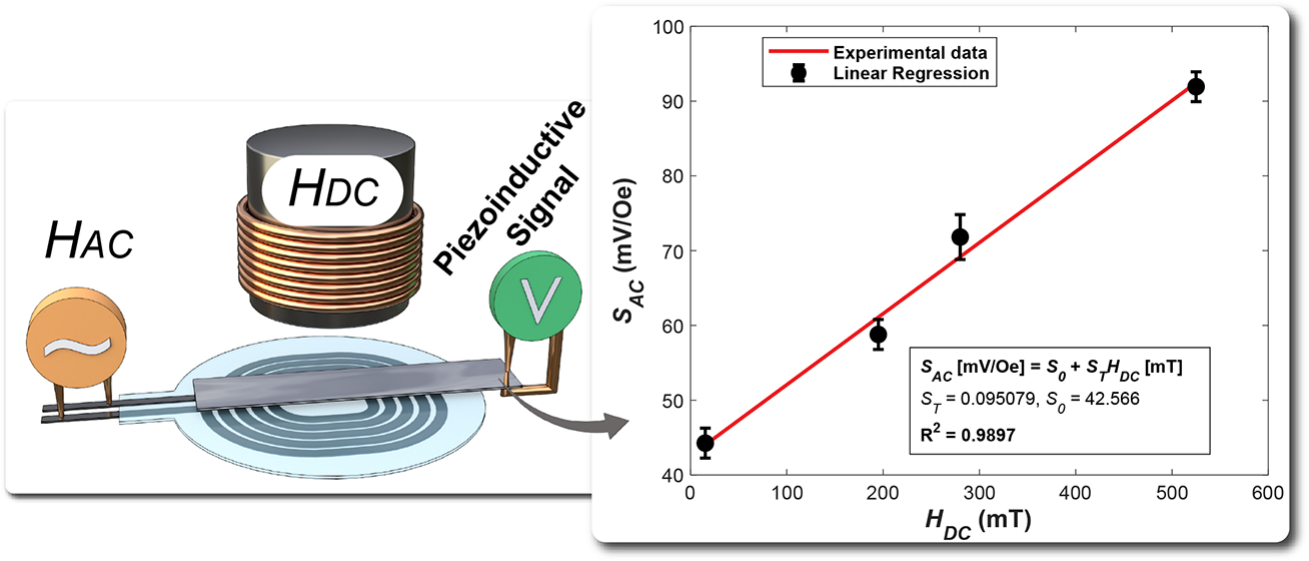

Additive manufacturing (AM) is emerging as an eco-friendly method for minimizing waste, as the demand for responsive materials in IoT and Industry 4.0 is on the rise. Magnetoactive composites, which are manufactured through AM, facilitate nonintrusive remote sensing and actuation. Printed magnetoelectric composites are an innovative method that utilizes the synergies between magnetic and electric properties. The study of magnetoelectric effects, including the recently validated piezoinductive effect, demonstrates the generation of electric voltage through external AC and DC magnetic fields. This shift in magnetic sensors, utilizing piezoinductive effect of the piezoelectric polymer poly(vinylidene fluoride), PVDF, eliminates the need for magnetic fillers in printed devices, aligning with sustainability principles, essential for the deployment of IoT and Industry 4.0. The achieved sensitivity surpasses other studies by 100 times, showcasing linear outputs for both applied AC and DC magnetic fields. Additionally, the sensor capitalizes on the linear phase shift of the generated signal with an applied DC magnetic field, an unprecedented effect. Thus, this work introduces a remarkable magnetoactive device with a sensitivity of *S*_T_ = 95.1 ± 0.9 μV Oe^–1^ mT^–1^, a significantly improved performance compared to magnetoelectric devices using polymer composites. As a functional proof of concept of the developed system, a magnetic position sensor has been demonstrated.

## 1 Introduction

In the rapidly evolving landscape of the internet of things (IoT) and Industry 4.0, the demand for intelligent, responsive materials capable of sensing and adapting to dynamic environments has intensified.^1−4^ Within this framework, additive manufacturing (AM) stands as a foundation in the paradigm shift of Industry 4.0, offering a transformative approach to production that aligns with environmental responsibility.^5^ The inherent efficiency of this technology minimizes material waste, and it allows for precise, complex and customized layer-by-layer fabricated components, promoting design flexibility and functional and/or geometrical modifications.^6−8^ By enabling localized, on-demand production, AM significantly reduces the need for extensive transportation and storage, reducing associated carbon footprints.

Magnetoactive composites are essential components of the broader framework of multifunctional materials that are additively manufactured. These materials have the capacity to detect and react to external magnetic fields, which enables nonintrusive remote sensing and actuation.^9−11^ These composites typically consist of a polymeric matrix in which magnetic fillers are included.^12^ Depending on the intended application; the quantity, composition, size, and morphology of the fillers can be varied. Printed magnetoelectric composites are an innovative means of exploiting the connection between magnetic and electric properties within the broader context of magnetoactive composites.^13−15^

The magnetolectric effect can be achieved by utilizing multimaterial architectures that have a structure-dependent magnetic-mechanical-to-electrical conversion. This conversion is based on the induction effect caused by the mechanical movement of a magnetic material over a coil, which generates a voltage that is directly proportional to the movement of the magnetic material. This requires a pliable framework that can be compressed mechanically displacing the magnetic component.^16,17^

Magnetoelectric effects within composite heterostructures of magnetostrictive and piezoelectric materials have been subject to increasing investigation.^18^ When subjected to an alternating magnetic field, magnetostriction causes deformation in the magnetostrictive layer, which is then transmitted to the piezoelectric layer through mechanical coupling, leading to the generation of an electric voltage.^19^

Various alternative effects can be used to manipulate magnetic and electric fields within composite structures. Particularly noteworthy is the observation that the intensity of the direct magnetoelectric effect in ferromagnetic-piezoelectric structures varies with the flow of an alternating electric current through the ferromagnetic layer.^20−22^ This phenomenon can be replicated by substituting the layer with a nonmagnetic conductor layer that carries alternating current.^23^ In this scenario, the piezoelectric-metal structure generates an alternating electric field, influenced by both the Ampere force acting on the current under an applied DC magnetic field and the piezoelectric effect in the piezoelectric layer. This effect is referred to as piezoinductive effect and arises from the combination of electromagnetic induction and piezoelectricity.^24^

This approach involves the generation of electric voltage through the application of external AC and DC magnetic fields, showcasing the response of a conventional magnetoelectric system containing only a piezoelectric material with previously deposited electrodes. This eliminates the need for magnetic fillers in the production of printed devices designed for magnetic field detection. This method represents a significant change in the field of magnetic sensors reliant on the magnetoelectric effect, introducing a more sustainable approach to device fabrication. Making use of AM capabilities while minimizing the need for magnetic fillers, leading to significant reductions in material waste.^25^

This effect has been recently experimentally validated in various configurations, including a radially poled lead zirconate titanate (PZT) ceramic ring,^26^ a PZT disk polarized perpendicular to the disk surface,^22^ and a unimorph bender constructed with PZT and alumina.^23^ Additionally, the phenomenon has been briefly observed in polyvinylidene fluoride (PVDF).^27,28^ Notably, the exploration of this effect has predominantly been focused on piezoelectric ceramic materials, with limited attention to materials beneficial to AM, such as PVDF.^29^ Furthermore, up until now, no published study has conducted a thorough analysis of the dielectric properties of the materials under investigation and how this affects material output, including phase shifts of the induced signal with applied DC magnetic field, as presented in this study.

This article establishes the groundwork for creating a compact AC/DC magnetic field sensor that relies on the piezoinductive effect of PVDF, utilizing both the voltage and the phase shift produced by the material under the application of AC magnetic fields created by a fully printed planar spiral inductor. A magnetic position sensor was also developed as proof of concept. The presented magnetoactive device is remarkable for presenting a notably superior response to magnetoelectric devices^30−33^ based on polymers without the need of fillers and being actuated by fully printed AC coil, making it suitable for AM and aligning with the sustainable principles integral to the future of IoT and Industry 4.0.

## 2 Materials and Methods

### 2.1 Materials

CuNi sputtered (80 nm) and polarized polyvinylidene difluoride (PVDF) with a thickness of 110 μm, *d*_33_ > 30 pC N^–1^, *d*_31_ > 30 pC N^–1^, dielectric constant ε ≈ 12.5 and Young modulus *E* > 2500 MPa was purchased from polyK. Dupont 5065 with a viscosity of 12–27 Pa s and resistivity ρ < 0.25 μΩ sq^–1^ was used as conductive silver ink for planar spiral inductor printing.

### 2.2 Fabrication of the Printed Spiral Inductor

**Figure 1:**
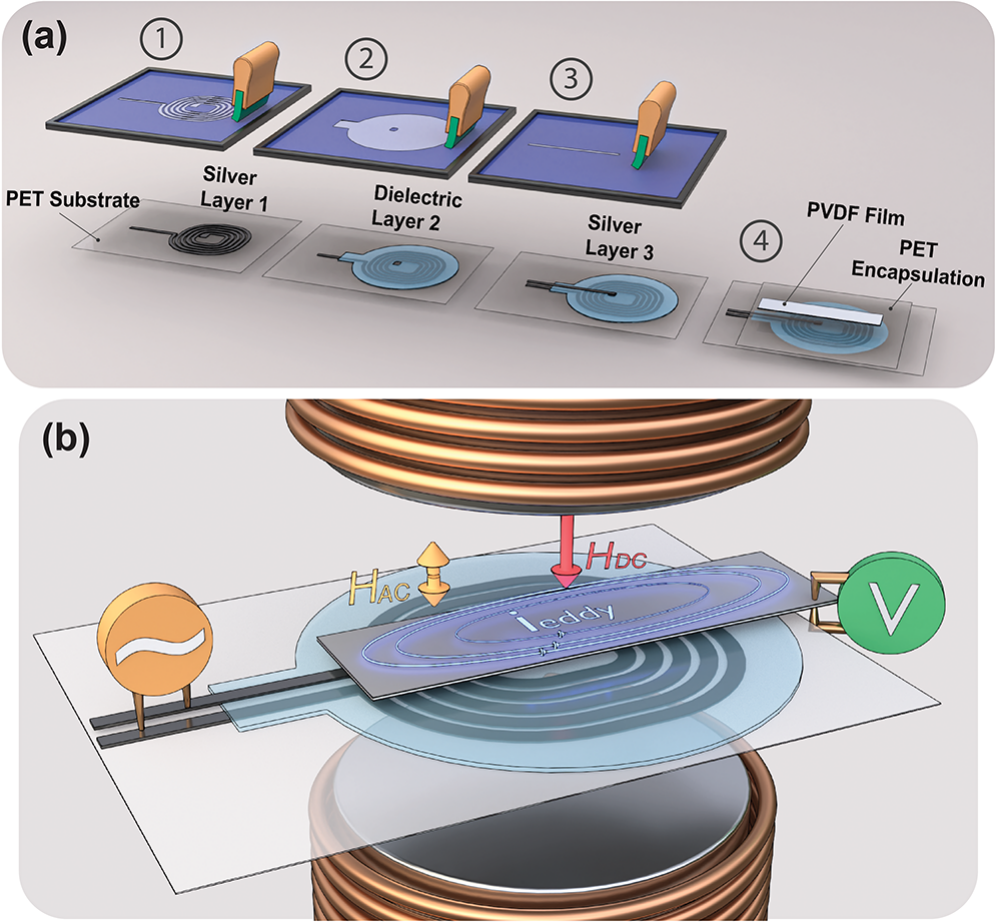
(a) Schematic diagram of the printing process and PVDF assembly. (b) Schematic representation of piezoelectric PVDF under AC and DC magnetic fields *H*_AC_ and *H*_DC_, respectively. *H*_AC_ is generated by the printed planar spiral coil, *H*_DC_, on the contrary, by an electromagnet.

The spiral inductor (5 turns, 1500 μm width and a spacing of 700 μm) was fabricated through a screen-printing method using a semiautomatic printer (model DX-3050D from DSTAR). The printing process utilized a vacuum table and an adjustable-speed printing squeegee ruler. The designed patterns were printed in multiple layers using a stencil with a frame dimension of 550 × 450 mm, positioned 3 mm away from the substrate. The printing speed was set at 30 cm s^–1^.^34^ A polyester screen with a mesh size of 195 × 195 threads per square inch (Dupont Teijin Melinex 506 PET substrate) was employed. The screen had an opening of 82 μm and a thickness of 89 μm, resulting in a theoretical ink volume of 35 g m^–2^. The mesh tension was maintained at 20 N during the printing process (Figure 1a). The conductive ink was cured at 80 °C during 1 h on an electric convection oven (JP selecta 2005165). The dielectric ink was cured with a UV Led of 405 nm during 10 min.

### 2.3 Electrical Characterization

Electromechanical response was characterized using a Quadtech 1920 LCR precision meter. Parallel capacitance (*C*_p_) and resistance (*R*_p_) were measured in order to determine PVDFs electromechanical resonance frequency. Furthermore, samples impedance (*Z*) and phase (ϕ) were measured too. All LCR measurements were performed at room temperature and at an applied voltage of 1 V.

### 2.4 Piezoinductive Measurements

All examined commercial PVDF samples were cut into 0.75 × 3 cm rectangles. The specimens were precisely positioned over a screen-printed planar spiral inductor, responsible for producing the AC magnetic field. These two components were strategically placed between the poles of an electromagnet (EMU-75), ensuring that the resulting DC magnetic field intersected perpendicularly with the sample (Figure S2). In this way, the sample experienced the influence of both an AC and DC magnetic field perpendicular to the PVDF surface. The AC magnetic field was generated by introducing a sinusoidal signal from the waveform generator (Rigol DG4202) into the printed coil. The resulting AC signal produced by the PVDF was captured by an oscilloscope (PicoScope 2205a). Subsequently, root-mean-square (rms) voltage data and phase shifts with respect to the signal injected into the planar spiral inductor, were directly extracted. The repeatability of the device was evaluated by performing multiple scans (at least 5 measurements) at different AC and DC magnetic fields. The experimental setup facilitated the concurrent adjustment of both the frequency and intensity of the AC and DC fields, providing flexibility for experimental modifications when necessary.

## 3 Results and Discussion

As previously mentioned, a sensor utilizing the piezoinductive effect requires the presence of both AC and DC magnetic fields to operate. In this study, the AC magnetic field is produced by a screen-printed planar coil of 5 turns, width of 1.5 mm, spacing of 0.7 mm and (see Materials and Methods) with a series resistance of *R*_s_ = 20 Ω. The coil is capable of generating a maximum AC magnetic field *H*_AC_ = 175 mOe by applying a maximum current *I*_rms_ = 275 mA. The chosen current value and consequently, the magnetic field strength, is carefully selected to prevent overheating of the coil. Further details about the printed planar spiral inductor can be found in Supporting Information (Figure S1).

Positioned at the exact center of the printed coil, the sample ensures that the magnetic field passing through the PVDF maintains maximum homogeneity.^35^ Furthermore, both the coil and the sample are securely affixed between two poles of an electromagnet, subjecting them to a DC magnetic field (see Figure 1b). Consequently, the sample experiences the effect of both an AC and DC magnetic field, both oriented out-of-plane with respect to the PVDF surface.

### 3.1 Material Characterization

Prior to assessing the piezoinductive characteristics of the studied PVDF, an examination of its dielectric properties is essential. Piezoinductive effect stems from the interaction between piezoelectric features and electromagnetic induction. Specifically, the process unfolds as follows: the applied alternating magnetic field induces a variation in magnetic flux through the sample over time, leading to the generation of eddy currents at the sample contacts in accordance with Faraday’s law.^36^ When subjected to a DC magnetic field (*H*_DC_), Ampere forces are produced, with their magnitude increasing proportionally to *H*_DC_. Consequently, the voltage generated between the two electrodes satisfy the following expression:^24,28,37^
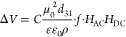
1where *C* is a constant that depends on the dimensions of the sample, μ_0_ and ε_0_ are the permeability and permittivity in vacuum respectively, *d*_31_ is the transverse piezoelectric coefficient, ε the permittivity of the PVDF, ρ the resistivity of the electrodes and *f* the frequency of the AC magnetic field applied to the sample.

This expression aligns with experimental observations in other studies, indicating a proportionality between the response and both the applied DC and AC magnetic fields.^24,27^ Furthermore, given the piezoelectric nature of the material and its electromechanical resonance frequency (*f*_r_), where the generated voltage reaches its peak, the piezoinductive response is maximized at *f*_r_. It is for that reason that it is essential to analyze the dielectric properties, since by means of capacitance (*C*_p_) and parallel resistances (*R*_p_) and impedance (*Z*) and phase (ϕ) measurements, *f*_r_ can be determined and the sensor response can be maximized.

**Figure 2:**
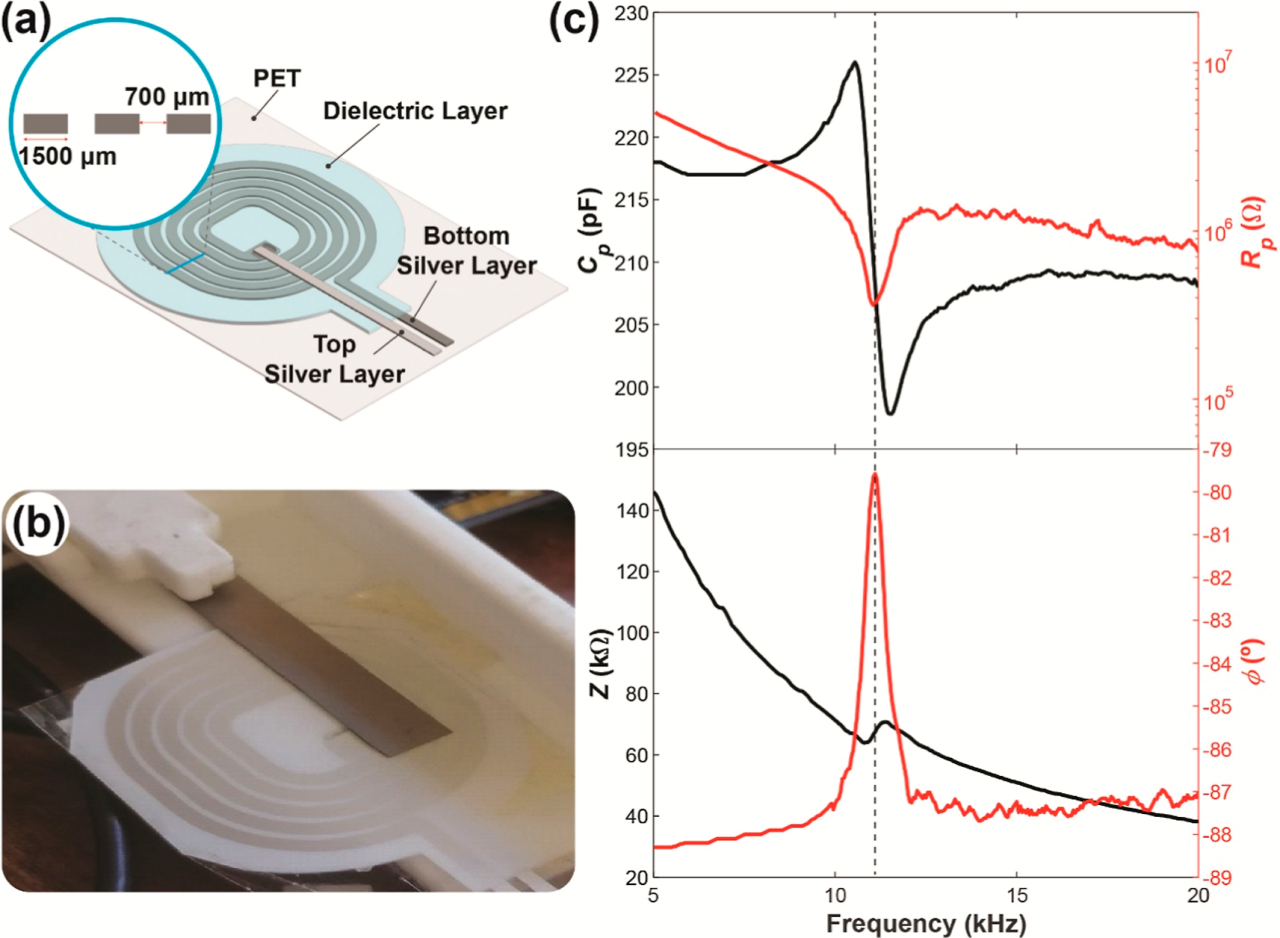
(a) Schematic representation of the printed planar spiral coils printing layers; (b) samples position atop the printed coil; (c) frequency response of PVDFs parallel capacitance (*C*_p_), resistance (*R*_p_), impedance (*Z*) and phase (ϕ).

Figure 2c illustrates the dielectric response of the examined PVDF. Notably, all studied samples exhibit a consistent resonance frequency at *f*_r_ = 10.9 ± 0.2 kHz. At this frequency, a marked decrease in *R*_p_ occurs, accompanied by a characteristic electromechanical resonance at *C*_p_, typical in piezoelectric materials.^24^ In contrast to ceramic piezoelectrics like PZT, where resonances and antiresonances with a notable quality factor are generally observed, the sample does not display sharp resonances in *Z*. While transitions in ϕ from positive to negative values are common in ceramic piezoelectrics, resulting in resonances or antiresonances, the PVDF consistently maintains negative ϕ values. Despite this, ϕ exhibits a maximum at the same frequency as *f*_r_. Corresponding to this maximum in ϕ, the impedance experiences a damped resonance, as observed in previous works.^38,39^ Specifically, the impedance slightly decreases just below *f*_r_ and slightly increases just above *f*_r_. This damping is attributed to the substantial mechanical losses characteristic of these materials compared to inorganic piezoelectrics.^39^ Additionally, the phase always remains negative, a phenomenon associated with mechanical losses.^38^ These effects, known and previously observed in experimental works, distinguish the PVDF behavior from that of inorganic piezoelectrics.

### 3.2 Piezoinductive Response

**Figure 3:**
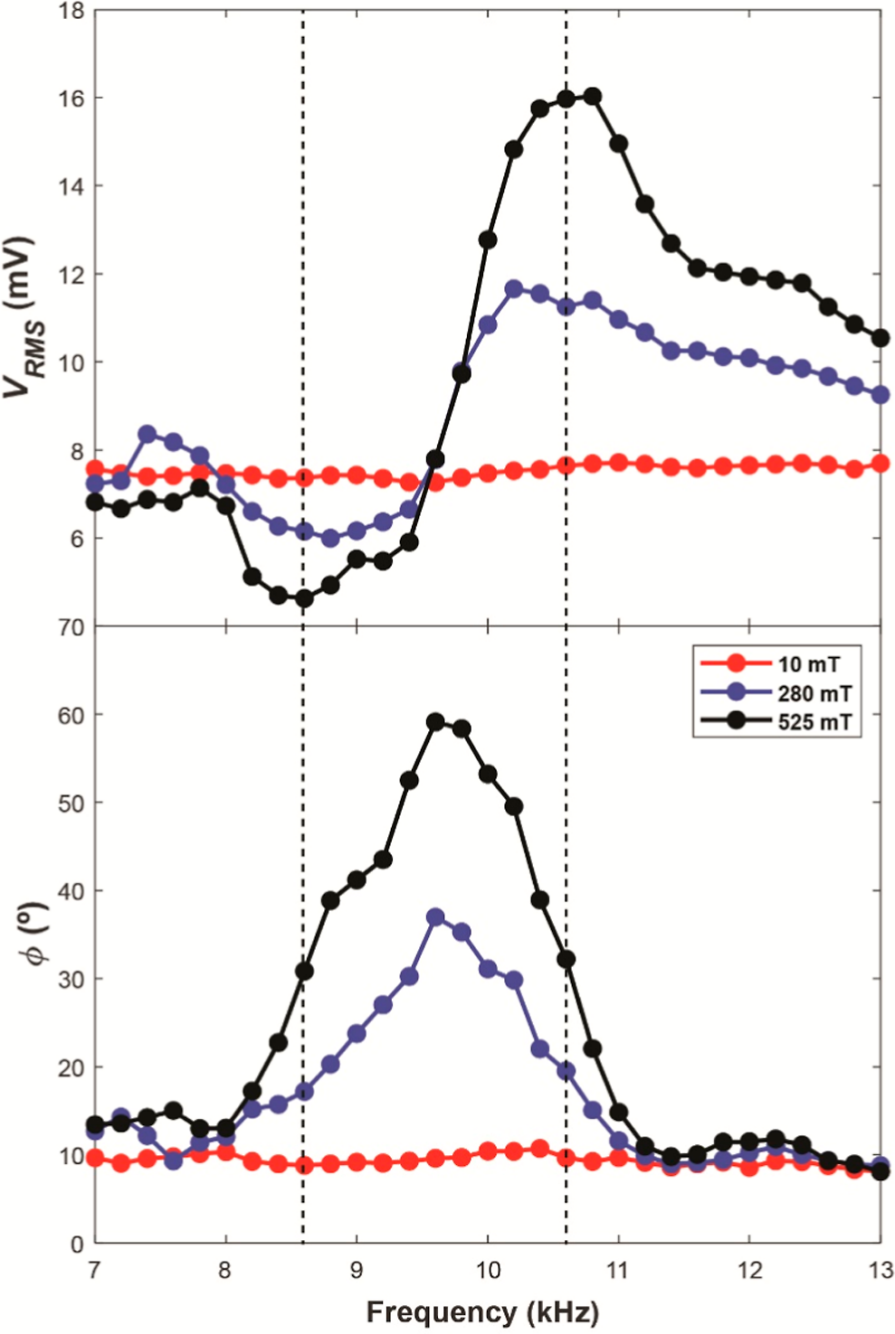
Effect of applied DC magnetic field on the rms voltage and phase of the signal generated by the PVDF sample under a constant AC magnetic field intensity *H*_AC_ = 175 mOe.

After analyzing the dielectric response of the studied PVDF and determining its electromechanical resonance frequency, the piezoinductive properties can be thoroughly examined. As previously stated, the piezoinductive response primarily relies on the frequency of the AC magnetic field and the intensity of the *H*_AC_ and *H*_DC_ magnetic fields. In Figure 3, the effect of the applied *H*_DC_ on the voltage generated by the samples at various frequencies is illustrated. It is shown that the rms voltage profile (*V*_rms_) produced by the PVDF closely mirrors the previously analyzed *Z* profile (see Figure 2c), although shifted 1.3 kHz toward more negative frequencies. This shift is attributed to the inductive coupling between the printed coil, responsible for applying the AC magnetic field, and the PVDF sample (Figure S3). Two resonances, corresponding to the inflection points of the phase of the signal generated by the PVDF, increase in magnitude with increasing *H*_DC_. The resonance at the highest frequency, *f* = 10.6 kHz, exhibits the greatest amplitude, justifying its selection as the operating frequency for the proposed device in this study.

**Figure 4:**
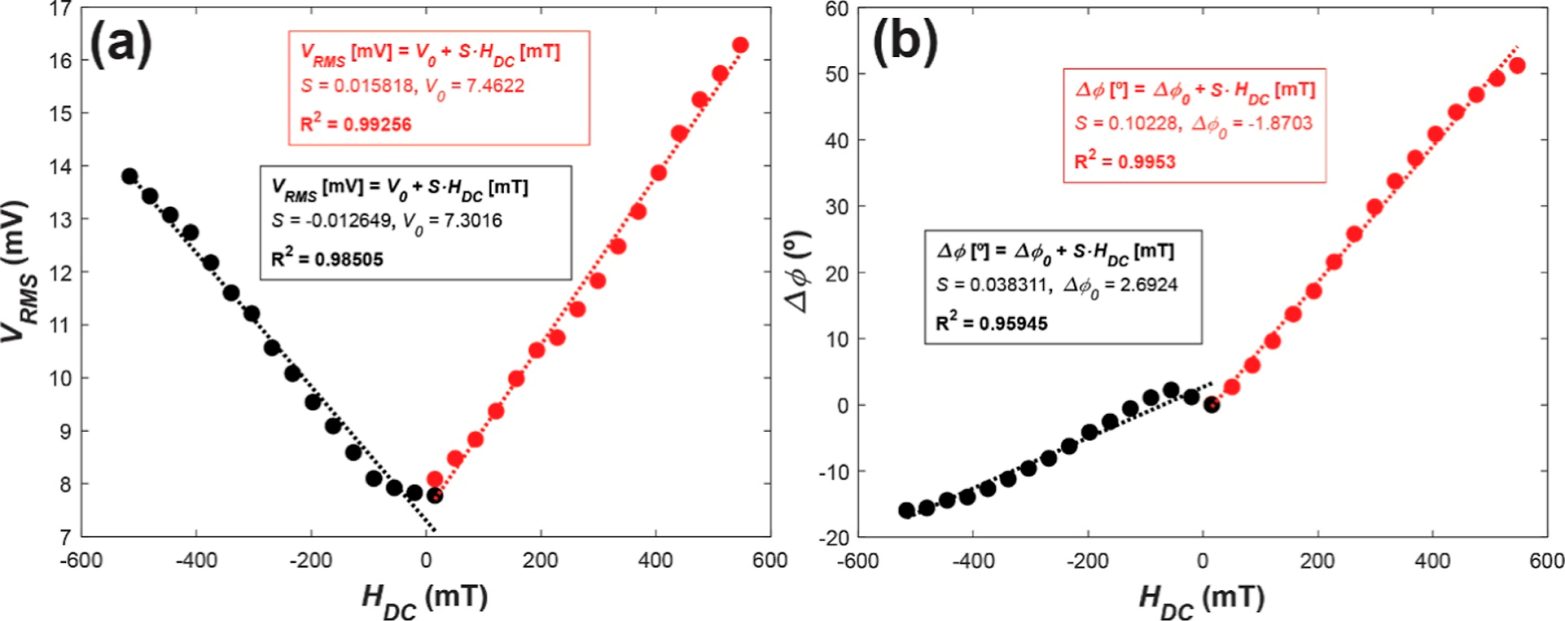
Linear dependence of both the (a) rms voltage and (b) phase shift with respect to the applied DC magnetic field under a constant AC magnetic field intensity *H*_AC_ = 175 mOe at a frequency *f* = 10.6 kHz.

After establishing the operational frequency of the PVDF-based device, the sample responses were calibrated by modulating both *H*_DC_ and *H*_AC_ values. Concerning the effect of *H*_DC_, it was observed that *V*_rms_ increases in a linear fashion with the applied DC magnetic field, demonstrating a sensitivity of *S*_DC_ = 14.24 ± 2.21 μV·mT^–1^ for positive and negative *H*_DC_. The presence of residual magnetism in the magnetic core of the electromagnet can explain the small variation of *S*_DC_ for positive and negative *H*_DC_ (Figure 4a). Additionally, the signal generated by the piezoelectric PVDF undergoes a linear phase shift relative to the signal injected into the printed coil. This shift is positive for positive magnetic fields and negative for negative magnetic fields. This characteristic enables the device to function as a potential AC/DC magnetic field sensor, as the observed phase shift (Figure 4b) can be used to determine the polarity of the measured DC magnetic field. Figure S4 shows the results regarding the PVDF and a PZT disc regarding the phase. In the case of the PZT disc, the phase shift is immediate at the resonance frequency, changing from negative to positive, passing through zero. As for the PVDF polymer, the linear response in the phase is related to the lower piezoelectric coefficient and higher impedance of the PVDF material that does not allow immediate change in the phase. A typical electronic conditioning setup for a piezoelectric transducer involves a charge amplifier that filters and amplifies its response.^40^ For the material characterized in this study, it is suggested to incorporate a phase detector capable of discerning whether the signal is delayed or advanced concerning a reference signal; in this case, the signal applied to the printed coil.

**Figure 5:**
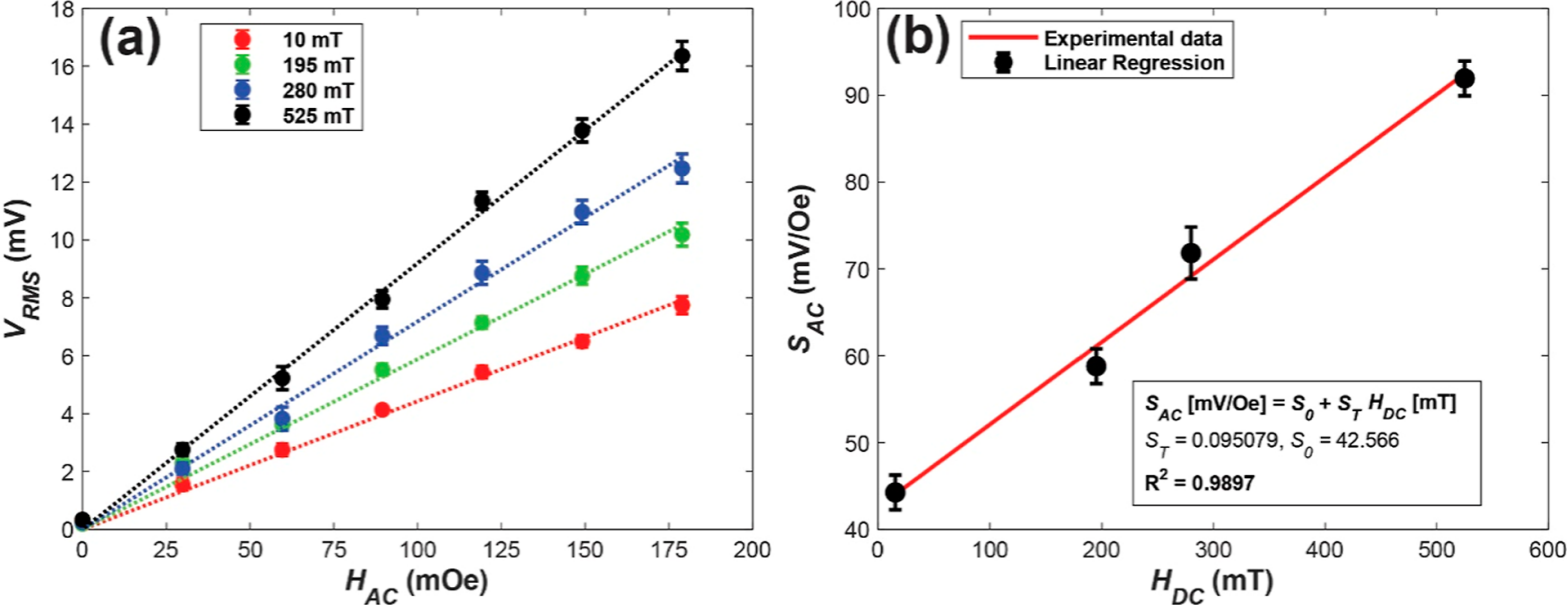
Linear dependence of (a) the rms voltage generated by the PVDF sample with applied AC magnetic field intensity at a frequency *f* = 10.6 kHz and different DC magnetic fields, provided in the inset; and (b) the AC sensitivity with respect to the applied DC magnetic field.

Figure 5a shows how the *V*_rms_ voltage of the device also shows a linear dependence with *H*_AC_, consistent with the previous theoretical predictions. This outcome validates the proportional relationship between the voltage generated by the piezoelectric material and both *H*_AC_ and *H*_DC_, as indicated by eq 1. As the applied DC magnetic field increases, the linearity of the response to *H*_AC_ is consistently maintained. This observation is intriguing because, a device based on the direct magnetoelectric effect typically increases the generated voltage as *H*_DC_ approaches the coercive field of the magnetostrictive material and decreases as *H*_DC_ moves away from that same coercive field.^41^ This reflects a nonlinear behavior of the device with respect to the applied DC magnetic field, which limits its applicability. Given the electromagnetic induction basis, the sensitivity of an ideal piezoinductive device, on the contrary, would theoretically linearly increase infinitely with *H*_DC_.^42,43^ While the real case may not reach infinity, the response would continue to increase up to the mechanical limit of the PVDF. That is why the dependence of the AC sensitivity (*S*_AC_) on *H*_DC_ has also been studied.

In Figure 5b, the various AC sensitivities corresponding to each DC magnetic field are plotted. As anticipated from eq 1, it is observed that *S*_AC_ exhibits a linear increase with *H*_DC_. Unfortunately, consistent with the findings in Figure 3, where the baseline *V*_rms_ amplitude of the signal generated by the samples is approximately 7.5 mV, the linear fit does not intersect values close to 0 on the *Y*-axis. This suggests that under the influence of any DC magnetic field, a voltage difference is expected to be generated. It becomes apparent that eq 1 should include not only a term directly proportional to *H*_DC_ but also an additional term dependent on *H*_AC_. Thus, the following new relation for the generated voltage (ΔV*) is proposed
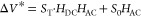
2where *S*_T_ represents the overall sensitivity, considering both *H*_AC_ and *H*_DC_, with units V Oe^–1^ mT^–1^, and *S*_0_ denotes the baseline induction in the sample, measured in units of V Oe^–1^. For a detailed account of the development leading to eq 2, see Supporting Information (Section D). When applying the formula to the data derived from the linear fit in Figure 5b and comparing it with the experimental data in Figure 4a, a maximum relative error of 3% has been calculated for *H*_DC_ values of 525 mT. In the literature, there is no agreement when determining the figures of merit of these devices, sometimes the voltage generated by the device is normalized with respect to both *H*_AC_ and *H*_DC_ (equivalent to *S*_T_) and sometimes only with respect to *H*_DC_ or *H*_AC_. Additionally, many studies omit the baseline induction component at zero DC magnetic field (*S*_0_), likely because works has been predominantly focused on ceramic materials under highly uniform AC magnetic fields. However, given that the device in this work is based on a polymeric material and that the uniformity of the magnetic field generated by a printed planar spiral coil is lower than that of a Helmholtz coil, *S*_0_ is no longer negligible. That is why the definition proposed in eq 2 proves to be more precise and practical, as it diminishes the theoretical response of the piezoinductive device to *S*_T_ and *S*_0_, both of which are experimentally measurable parameters through sweeps in *H*_AC_ and *H*_DC_.

As the operational principle of the device proposed in this work is identical to that of other published piezoinductive devices and even with other magnetoelectric devices, it is instructive to draw some comparisons between our work and such devices (Table 1).

**Table 1:** Polymeric and Ceramic Piezoinductive and Magnetoelectric Figures of Merit Found in Literature

materials	effect	geometry	*S*_T_ [mV Oe^–1^ mT^–1^]	*S*_0_ [mV Oe^–1^]	α_max_ [V cm^–1^ Oe^–1^]	refs
PZT/Ni	direct magnetoelectric	ring			2.440	(20)
P(VDF–TrFE) + Fe_72.5_Si_12.5_B_15_	direct magnetoelectric	rectangle			0.065	(44)
P(VDF–TrFE) + CoFe_2_O_4_	direct magnetoelectric	rectangle			0.041	(41)
P(VDF–TrFE) + CoFe_2_O_4_	direct magnetoelectric	rectangle			0.021	(45)
P(VDF–TrFE)/PVDF + CoFe_2_O_4_	direct magnetoelectric	rectangle			0.164	(46)
PZT/Ag	piezoinductive	ring	0.16	≈0	0.64	(26)
PZT/Ag	piezoinductive	disk	3.208	≈0	≈80	(24)
P(VDF–TrFE) + Fe_3_O_4_	piezoinductive	disk	0.0019	0.144	0.089	(37)
PVDF/Ag	piezoinductive	disk	0.010	0.001	0.384	(27)
PVDF/Ag	piezoinductive	rectangle	0.003	0.001	0.299	(28)
PVDF/CuNi	piezoinductive	rectangle	0.095	43.566	8.357	this work

Taking into account that the PVDF samples have a thickness of 110 μm, maximum values of α_33_ = 8.357 V cm^–1^ Oe^–1^ at 525 mT have been obtained, values even 100 times higher than others obtained in other works based on PVDF.^27,28,37^ Moreover, the magnetoelectric coefficient is only 1 order of magnitude below those obtained in devices based on ceramic piezoelectrics such as PZT.^23,24,26^ The high sensitivity achieved by the proposed device without the need for magnetic fillers in the piezoelectric material, differentiates the current work from prior magnetoelectric studies. The developed sensor can be used, among other applications, as magnetic position sensors, where the amplitude of the signal generated by the device will change decrease or increase depending on how close or far it is from a permanent magnet.

### 3.3 Proof of Concept of a Magnetic Position Sensor

**Figure 6:**
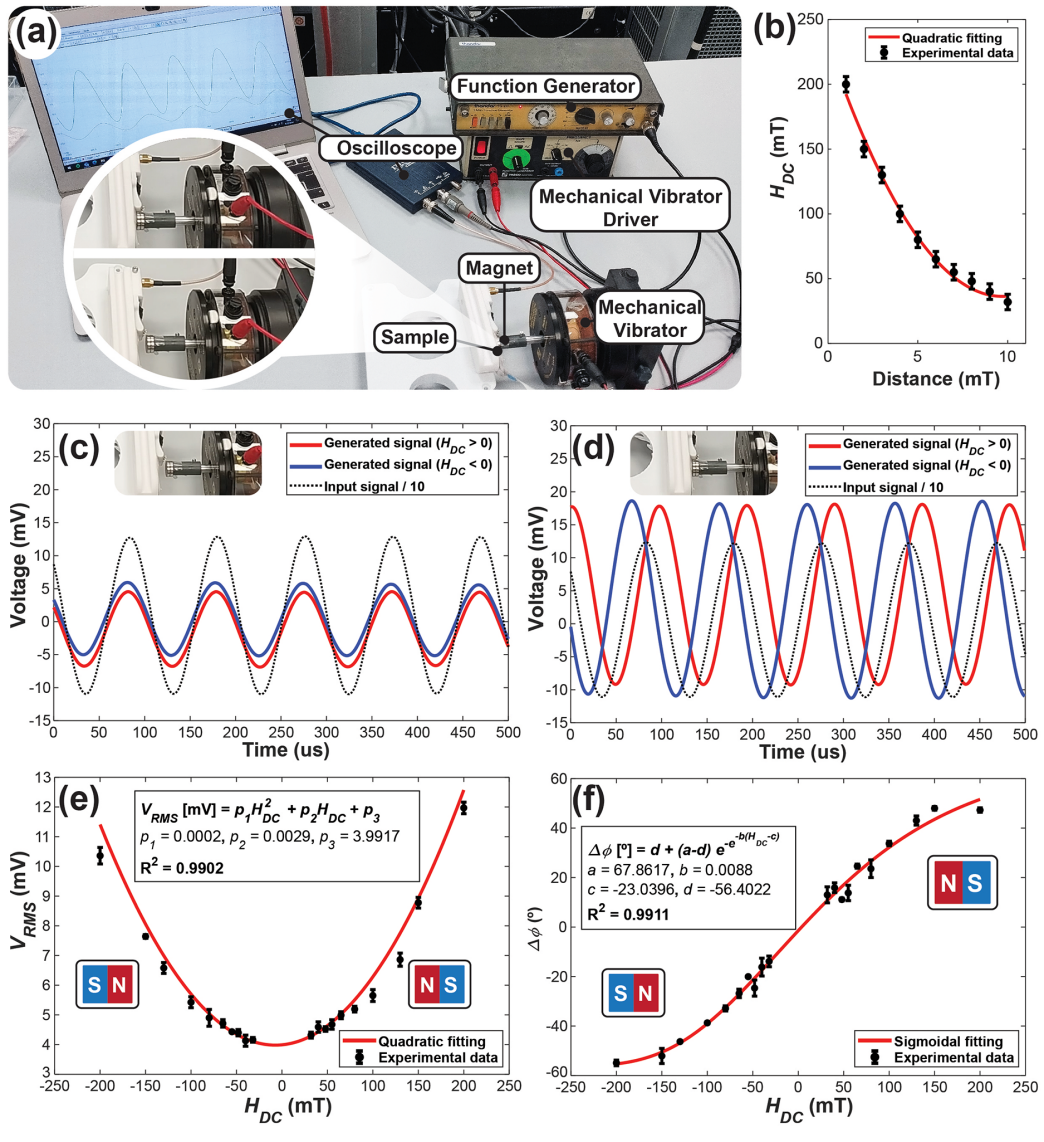
(a) Photograph of the position sensor configuration, (b) magnetic field measurements as a function of distance from the sensor. The voltage generated by the piezoinductive sensor was measured for both NS and SN configurations with the magnet positioned at distances of 10 (c) and 1 mm (d). (e) Sensitivity of the sensor as a function of the magnetic field, (f) Phase shift as a function of the magnetic field.

Based on the characteristics of the developed sensor, an application as a magnetic position sensor was demonstrated. The piezoinductive sensor was placed at close proximity with a magnet (15 mm diameter by 3 mm thickness, grade N52, from Supermagnete). The magnet was attached to a mechanical vibrator controlled by a function generator at a frequency of 1 Hz. This enables a distance variation of the magnet with respect to the sensor, thus modulating the DC magnetic field intensity *H*_DC_ applied to the piezoinductive sensor. The sensors printed coil is connected to another function generator creating the *H*_AC_, and the PVDF sample is connected to the oscilloscope in order to visualize the generated signal variations (Figure 6 and Supporting Information Movie S1). The magnetic field sensed by the sensor was measured by a gaussmeter (GM08 from Hirst Magnetics).

Figure 6 shows the results of the developed position sensor when the magnet is brought within 10 mm of the sensor at a frequency of 1 Hz. To eliminate oscillations caused by touch or air movement, the magnet was positioned at a distance of 1 mm from the sensor at its closest approach. The frequency was kept constant because the mechanical vibrator adjusts the piston distance based on the frequency: the lower the frequency, the further the piston moves. The magnetic field sensed by the device varies from 200 to 28 mT (Figure 6b). The magnet polarization direction was inverted (NS to SN) in order to analyze the phase shift in the signal. Figure 6c,d shows the phase shift of the signal from the sensor compared with the input signal, the signal shifting to the right side for positive *H*_DC_ and in the opposite direction for negative *H*_DC_, allowing the detection of the direction of the magnetic field by the phase shift. The amplitude of the signal for positive and negative fields was analyzed in Figure 6e, where the symmetry of the magnetic field’s behavior as a function of distance was verified. When comparing the results from Figures 6f with 4b, the phase shift utilizing the magnet exhibits a more symmetrical behavior of the phase. The asymmetry observed in Figure 4b can be attributed to some remanence in the magnetic core of the electromagnet, which diminishes the sensor ability to reach a higher value.

Incorporating a band-pass filter can mitigate the high frequency noise, as well as the low frequency noise caused by the pyroelectric effect of the PVDF, background frequencies and sensor movement.

## 4 Conclusions

This work establishes the groundwork for the development of a compact AC/DC magnetic field sensor that relies on the piezoinductive effect of PVDF, utilizing both the voltage and the phase shift produced by the material under the application of AC magnetic fields created by a fully printed planar spiral inductor. The presented magnetoactive device is remarkable for presenting an overall sensitivity of *S*_T_ = 95.1 ± 0.9 μV Oe^–1^ mT^–1^, a notably superior response to magnetoelectric devices based on polymer composites without the need of magnetic fillers and being actuated by fully printed AC coil, making it suitable for AM fabrication and aligning with the sustainable principles integral to the future of IoT and Industry 4.0. The applicability of the system has been shown by developing a magnetic position sensor.

## Supporting Information Available

Screen-printed planar spiral coil calibration, radiation patterns and field lines; experimental piezoinductive measurements setup; resonance shift via inductive coupling between printed coil and piezoelectric PVDF sample; formulation of the generated voltage based on experimental AC sensitivity measurements (PDF)
Visualize the generated signal variations (MP4)

## References

[ref1] Qiu X.; Bian Y.; Mei X.; Luo X.; Hu Z.; Xuan F.-Z.; Xiang Y.; Zhu G. Fully Transparent Flexible Piezoelectric Sensing Materials Based on Electrospun PVDF and Their Device Applications. Adv. Mater. Technol. 2024, 9, 2301494 10.1002/admt.202301494.

[ref2] Akhter F.; Siddiquei H. R.; Alahi M. E. E.; Jayasundera K. P.; Mukhopadhyay S. C. An IoT-Enabled Portable Water Quality Monitoring System With MWCNT/PDMS Multifunctional Sensor for Agricultural Applications. IEEE Internet Things J. 2022, 9 (16), 14307–14316. 10.1109/jiot.2021.3069894.

[ref3] Kumar A.; Preeti Km.; Singh S. P.; Lee S.; Kaushik A.; Sharma S. K. ZnO-Based Hybrid Nanocomposite for High-Performance Resistive Switching Devices: Way to Smart Electronic Synapses. Mater. Today 2023, 69, 262–286. 10.1016/j.mattod.2023.09.003.

[ref4] Choi H. W.; Shin D.-W.; Yang J.; Lee S.; Figueiredo C.; Sinopoli S.; Ullrich K.; Jovančić P.; Marrani A.; Momentè R.; Gomes J.; Branquinho R.; Emanuele U.; Lee H.; Bang S. Y.; Jung S.-M.; Han S. D.; Zhan S.; Harden-Chaters W.; Suh Y.-H.; Fan X.-B.; Lee T. H.; Chowdhury M.; Choi Y.; Nicotera S.; Torchia A.; Moncunill F. M.; Candel V. G.; Durães N.; Chang K.; Cho S.; Kim C.-H.; Lucassen M.; Nejim A.; Jiménez D.; Springer M.; Lee Y.-W.; Cha S.; Sohn J. I.; Igreja R.; Song K.; Barquinha P.; Martins R.; Amaratunga G. A. J.; Occhipinti L. G.; Chhowalla M.; Kim J. M. Smart Textile Lighting/Display System with Multifunctional Fibre Devices for Large Scale Smart Home and IoT Applications. Nat. Commun. 2022, 13 (1), 814 10.1038/s41467-022-28459-6. 35145096 PMC8831553

[ref5] Hegab H.; Khanna N.; Monib N.; Salem A. Design for Sustainable Additive Manufacturing: A Review. Sustainable Mater. Technol. 2023, 35, e00576 10.1016/j.susmat.2023.e00576.

[ref6] Chen Q.; Zhao J.; Ren J.; Rong L.; Cao P.-F.; Advincula R. C. 3D Printed Multifunctional, Hyperelastic Silicone Rubber Foam. Adv. Funct. Mater. 2019, 29 (23), 1900469 10.1002/adfm.201900469.

[ref7] Lee Y.-W.; Kim J.-K.; Bozuyuk U.; Dogan N. O.; Khan M. T. A.; Shiva A.; Wild A.-M.; Sitti M. Multifunctional 3D-Printed Pollen Grain-Inspired Hydrogel Microrobots for On-Demand Anchoring and Cargo Delivery. Adv. Mater. 2023, 35 (10), 2209812 10.1002/adma.202209812. 36585849

[ref8] Jiang Y.; Islam M. N.; He R.; Huang X.; Cao P.-F.; Advincula R. C.; Dahotre N.; Dong P.; Wu H. F.; Choi W. Recent Advances in 3D Printed Sensors: Materials, Design, and Manufacturing. Adv. Mater. Technol. 2023, 8 (2), 2200492 10.1002/admt.202200492.

[ref9] Shlapakova L. E.; Botvin V. V.; Mukhortova Y. R.; Zharkova I. I.; Alipkina S. I.; Zeltzer A.; Dudun A. A.; Makhina T.; Bonartseva G. A.; Voinova V. V.; Wagner D. V.; Pariy I.; Bonartsev A. P.; Surmenev R. A.; Surmeneva M. A. Magnetoactive Composite Conduits Based on Poly(3-Hydroxybutyrate) and Magnetite Nanoparticles for Repair of Peripheral Nerve Injury. ACS Appl. Bio Mater. 2024, 7 (2), 1095–1114. 10.1021/acsabm.3c01032. 38270084

[ref10] Wajahat M.; Kim J. H.; Kim J. H.; Jung I. D.; Pyo J.; Seol S. K. 4D Printing of Ultrastretchable Magnetoactive Soft Material Architectures for Soft Actuators. ACS Appl. Mater. Interfaces 2023, 15 (51), 59582–59591. 10.1021/acsami.3c12173. 38100363

[ref11] Zhang Z.; Heron J. T.; Pena-Francesch A. Adaptive Magnetoactive Soft Composites for Modular and Reconfigurable Actuators. Adv. Funct. Mater. 2023, 33 (26), 2215248 10.1002/adfm.202215248.

[ref12] Díez A. G.; Tubio C. R.; Gómez A.; Berastegi J.; Bou-Ali M. M.; Etxebarria J. G.; Lanceros-Mendez S. Tuning Magnetorheological Functional Response of Thermoplastic Elastomers by Varying Soft-Magnetic Nanofillers. Polym. Adv. Technol. 2022, 33 (8), 2610–2619. 10.1002/pat.5717.

[ref13] Wu H.; Luo R.; Li Z.; Tian Y.; Yuan J.; Su B.; Zhou K.; Yan C.; Shi Y. Additively Manufactured Flexible Liquid Metal–Coated Self-Powered Magnetoelectric Sensors with High Design Freedom. Adv. Mater. 2024, 36, 2307546 10.1002/adma.202307546. 38145802

[ref14] Brito-Pereira R.; Martins P.; Lanceros-Mendez S.; Ribeiro C. Polymer-Based Magnetoelectric Scaffolds for Wireless Bone Repair: The Fillers’ Effect on Extracellular Microenvironments. Compos. Sci. Technol. 2023, 243, 110263 10.1016/j.compscitech.2023.110263.

[ref15] Sasmal A.; Arockiarajan A. Recent Progress in Flexible Magnetoelectric Composites and Devices for next Generation Wearable Electronics. Nano Energy 2023, 115, 108733 10.1016/j.nanoen.2023.108733.

[ref16] Wu H.; Zhang X.; Ma Z.; Zhang C.; Ai J.; Chen P.; Yan C.; Su B.; Shi Y. A Material Combination Concept to Realize 4D Printed Products with Newly Emerging Property/Functionality. Adv. Sci. 2020, 7 (9), 1903208 10.1002/advs.201903208. PMC7201257 32382481

[ref17] Wu H.; Wang Q.; Wu Z.; Wang M.; Yang L.; Liu Z.; Wu S.; Su B.; Yan C.; Shi Y. Multi-Material Additively Manufactured Magnetoelectric Architectures with a Structure-Dependent Mechanical-to-Electrical Conversion Capability. Small Methods 2022, 6 (12), 2201127 10.1002/smtd.202201127. 36307387

[ref18] Nan C.-W.; Bichurin M. I.; Dong S.; Viehland D.; Srinivasan G. Multiferroic Magnetoelectric Composites: Historical Perspective, Status, and Future Directions. J. Appl. Phys. 2008, 103 (3), 031101 10.1063/1.2836410.

[ref19] Gutiérrez J.; Lasheras A.; Martins P.; Pereira N.; Barandiarán J.; Lanceros-Mendez S. Metallic Glass/PVDF Magnetoelectric Laminates for Resonant Sensors and Actuators: A Review. Sensors 2017, 17 (6), 1251 10.3390/s17061251. 28561784 PMC5492088

[ref20] Xu L.; Qiao L.; Pan D.; Volinsky A. A. Lorentz Force Induced Magnetoelectric Effect in Cylindrical Composites with PZT/Magnetostrictive and Nonmagnetic Metal Layers. J. Alloys Compd. 2018, 730, 483–486. 10.1016/j.jallcom.2017.09.211.

[ref21] Leung C. M.; Or S. W.; Ho S. L. Dc Magnetoelectric Sensor Based on Direct Coupling of Lorentz Force Effect in Aluminum Strip with Transverse Piezoelectric Effect in 0.7Pb(Mg1/3Nb2/3)O3–0.3PbTiO3 Single-Crystal Plate. J. Appl. Phys. 2010, 107 (9), 09E702 10.1063/1.3337748.

[ref22] Jia Y. M.; Zhou D.; Luo L. H.; Zhao X. Y.; Luo H. S.; Or S. W.; Chan H. L. W. Magnetoelectric Effect from the Direct Coupling of the Lorentz Force from a Brass Ring with Transverse Piezoelectricity in a Lead Zirconate Titanate (PZT) Disk. Appl. Phys. A: Mater. Sci. Process. 2007, 89 (4), 1025–1027. 10.1007/s00339-007-4209-0.

[ref23] Guiffard B.; Guyomar D.; Garbuio L.; Belouadah R.; Zhang J.; Cottinet P. J. Eddy Current Induced Magnetoelectricity in a Piezoelectric Unimorph Bender. Appl. Phys. Lett. 2010, 96 (4), 044105 10.1063/1.3298551.

[ref24] Fetisov Y. K. Piezoinductive Effect in Piezoelectric Disk With Electrodes Due to Combination of Electromagnetic Induction and Piezoelectricity. IEEE Sens. Lett. 2023, 7 (2), 1–4. 10.1109/lsens.2023.3240298.37529707

[ref25] Peng T.; Kellens K.; Tang R.; Chen C.; Chen G. Sustainability of Additive Manufacturing: An Overview on Its Energy Demand and Environmental Impact. Addit. Manuf. 2018, 21, 694–704. 10.1016/j.addma.2018.04.022.

[ref26] Fetisov Y. K.; Chashin D. V.; Srinivasan G. Piezoinductive Effects in a Piezoelectric Ring with Metal Electrodes. J. Appl. Phys. 2009, 106 (4), 044103 10.1063/1.3195073.

[ref27] Qi B.; Zhang Y.; Yao T.; Shang H. AC/DC Magnetic Field Sensing Utilizing Mechanically Mediated Product Effect of Ampere Force Caused by Eddy Currents and Piezoelectricity in a Magnetoelectric Disk. Phys. Status Solidi A 2021, 218 (17), 2000813 10.1002/pssa.202000813.

[ref28] Qi B.; Zhang Y.; Yao T. Magnetic Field Sensing Based on Magnetoelectric Coupling of Ampere Force Effect With Piezoelectric Effect in Silver/Poly(Vinylidine Fluoride)/Silver Laminated Composite. IEEE Access 2020, 8, 68049–68056. 10.1109/access.2020.2986174.

[ref29] Costa C. M.; Cardoso V. F.; Martins P.; Correia D. M.; Gonçalves R.; Costa P.; Correia V.; Ribeiro C.; Fernandes M. M.; Martins P. M.; Lanceros-Méndez S. Smart and Multifunctional Materials Based on Electroactive Poly(Vinylidene Fluoride): Recent Advances and Opportunities in Sensors, Actuators, Energy, Environmental, and Biomedical Applications. Chem. Rev. 2023, 123 (19), 11392–11487. 10.1021/acs.chemrev.3c00196. 37729110 PMC10571047

[ref30] Martins P.; Silva M.; Reis S.; Pereira N.; Amorín H.; Lanceros-Mendez S. Wide-Range Magnetoelectric Response on Hybrid Polymer Composites Based on Filler Type and Content. Polymers 2017, 9 (2), 62 10.3390/polym9020062. 30970740 PMC6432276

[ref31] Martins P.; Kolen’ko Yu. V.; Rivas J.; Lanceros-Mendez S. Tailored Magnetic and Magnetoelectric Responses of Polymer-Based Composites. ACS Appl. Mater. Interfaces 2015, 7 (27), 15017–15022. 10.1021/acsami.5b04102. 26110461

[ref32] Alnassar M.; Alfadhel A.; Ivanov Yu. P.; Kosel J. Magnetoelectric Polymer Nanocomposite for Flexible Electronics. J. Appl. Phys. 2015, 117 (17), 17D711 10.1063/1.4913943.

[ref33] Nan C.-W.; Cai N.; Shi Z.; Zhai J.; Liu G.; Lin Y. Large Magnetoelectric Response in Multiferroic Polymer-Based Composites. Phys. Rev. B: Condens. Matter Mater. Phys. 2005, 71 (1), 014102 10.1103/physrevb.71.014102.

[ref34] Gomes Correia V. M.; Pereira N.; Perinka N.; Costa P.; del Campo J.; Lanceros-Mendez S. Printed 3D Gesture Recognition Thermoformed Half Sphere Compatible with In-Mold Electronic Applications. Adv. Eng. Mater. 2022, 24 (12), 2200730 10.1002/adem.202200730.

[ref35] Pereira N.; Lima A. C.; Correia V.; Peřinka N.; Lanceros-Mendez S.; Martins P. Magnetic Proximity Sensor Based on Magnetoelectric Composites and Printed Coils. Materials 2020, 13 (7), 1729 10.3390/ma13071729. 32272728 PMC7212752

[ref36] Cullity B. D.; Graham C. D. Magnetization Dynamics. In Introduction to Magnetic Materials; John Wiley & Sons, Ltd, 2008; pp 409–438.

[ref37] Zhang J.-W.; Meng X.; Fu G.; Han T.; Xu F.; Putson C.; Liu X. Enhanced Polarization Effect of Flexible Magnetoelectric PVDF-TrFE/Fe3O4 Smart Nanocomposites for Nonvolatile Memory Application. ACS Appl. Electron. Mater. 2023, 5 (3), 1844–1852. 10.1021/acsaelm.3c00057.

[ref38] Ducrot P.-H.; Dufour I.; Ayela C. Optimization Of PVDF-TrFE Processing Conditions For The Fabrication Of Organic MEMS Resonators. Sci. Rep. 2016, 6 (1), 19426 10.1038/srep19426. 26792224 PMC4726305

[ref39] Ohigashi H. Electromechanical Properties of Polarized Polyvinylidene Fluoride Films as Studied by the Piezoelectric Resonance Method. J. Appl. Phys. 1976, 47 (3), 949–955. 10.1063/1.322685.

[ref40] Gonçalves S.; Serrado-Nunes J.; Oliveira J.; Pereira N.; Hilliou L.; Costa C. M.; Lanceros-Méndez S. Environmentally Friendly Printable Piezoelectric Inks and Their Application in the Development of All-Printed Touch Screens. ACS Appl. Electron. Mater. 2019, 1 (8), 1678–1687. 10.1021/acsaelm.9b00363.

[ref41] Martins P.; Lasheras A.; Gutierrez J.; Barandiaran J. M.; Orue I.; Lanceros-Mendez S. Optimizing Piezoelectric and Magnetoelectric Responses on CoFe2O4/P(VDF-TrFE) Nanocomposites. J. Phys. D: Appl. Phys. 2011, 44 (49), 495303 10.1088/0022-3727/44/49/495303.

[ref42] Magnetostatics. In Introduction to Electrodynamics; Griffiths D. J., Ed.; Cambridge University Press: Cambridge, 2017; pp 210–265.

[ref43] Cullity B. D.; Graham C. D. Definitions and Units. In Introduction to Magnetic Materials; John Wiley & Sons, Ltd, 2008; pp 1–21.

[ref44] Polícia R.; Lima A. C.; Pereira N.; Calle E.; Vázquez M.; Lanceros-Mendez S.; Martins P. Transparent Magnetoelectric Materials for Advanced Invisible Electronic Applications. Adv. Electron. Mater. 2019, 5 (12), 1900280 10.1002/aelm.201900280.

[ref45] Martins P.; Nunes J. S.; Oliveira J.; Peřinka N.; Lanceros-Mendez S. Spray-Printed Magnetoelectric Multifunctional Composites. Composites, Part B 2020, 187, 107829 10.1016/j.compositesb.2020.107829.

[ref46] Lima A. C.; Pereira N.; Policia R.; Ribeiro C.; Correia V.; Lanceros-Mendez S.; Martins P. All-Printed Multilayer Materials with Improved Magnetoelectric Response. J. Mater. Chem. C 2019, 7 (18), 5394–5400. 10.1039/c9tc01428d.

