# Supplementary material for: AC/DC Magnetic Field Sensing Based on a Piezoelectric Polymer and a Fully Printed Planar Spiral Coil
Source: ACS Appl Mater Interfaces. 2024 Aug 26;16(36):48547–55. doi: 10.1021/acsami.4c09409 (PMC11403604; doi:10.1021/acsami.4c09409)
Supplement: Supplementary file 1 — am4c09409_si_001.pdf [file am4c09409_si_001.pdf]

Supporting Information

AC/DC Magnetic Field Sensing Based on a Piezoelectric Polymer and a Fully Printed Planar Spiral Coil

Josu Fernández Maestu[a], *Nelson Pereira*[b]*, *Senentxu Lanceros-Méndez*[a,b,c]*

[a]*BCMaterials, Basque Center for Materials, Applications and Nanostructures, UPV/EHU Science Park, 48940, Leioa, Spain.*

[b]*Physics Center of Minho and Porto Universities (CF-UM-UP) and LaPMET - Laboratory of Physics for Materials and Emergent Technologies, University of Minho, 4710-057 Braga, Portugal.*

[c]*IKERBASQUE, Basque Foundation for Science, 48009, Bilbao, Spain.*

*E-mail: nelsonpereira@fisica.uminho.pt, senentxu.lanceros@bcmaterials.net

A. Screen-Printed Coil Calibration and Magnetic Field Lines

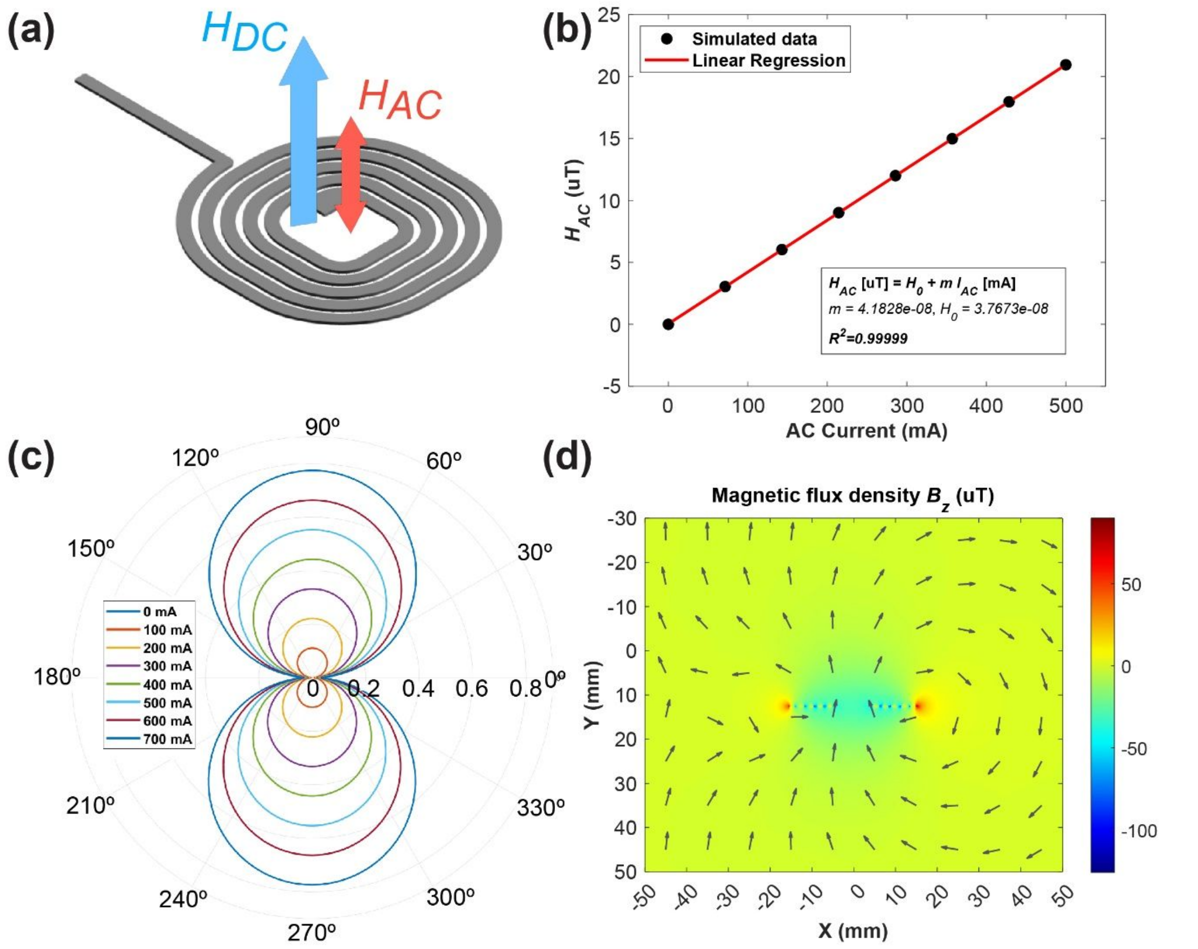

Figure S1. (a) Design and concept of the printed coil; (b) coil calibration; (c) far-field radiation patterns and (d) Z component magnetic flux density map (B_z) including magnetic field lines via finite element simulations.

B. Experimental piezoinductive measurements setup

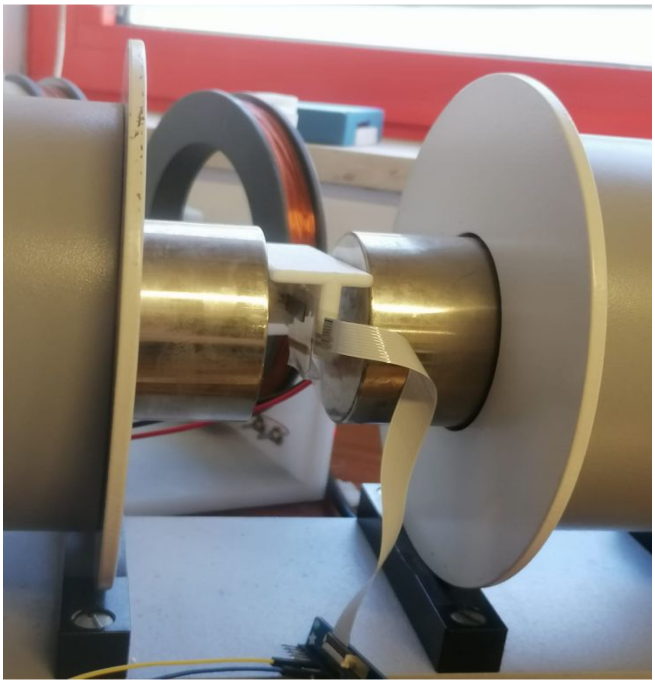

Figure S2. Experimental setup used to measure piezoinductive properties of PVDF samples. Samples are secured between electromagnets poles and placed atop the printed planar spiral coil.

C. Resonance shift via inductive coupling between printed coil and piezoelectric PVDF sample

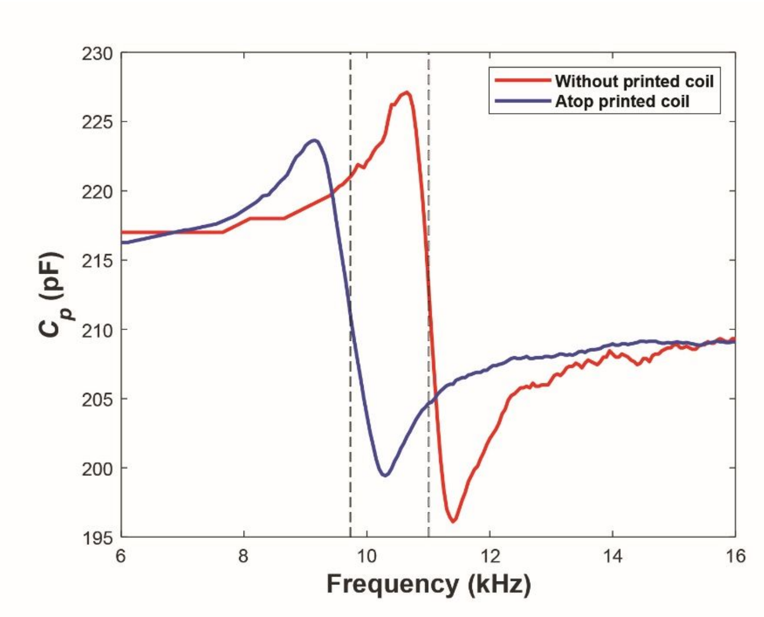

Figure S3. Effect of printed spiral planar coil on PVDFs electromechanical resonance.

D. Formulation of the generated voltage based on experimental AC sensitivity measurements

Based on the experimental results obtained for the AC sensitivity (S_{AC}) presented in **Figure 5** and keeping the excitation frequency constant, **Equation 1** has been reformulated in order to better represent the experimental results. Taking into account the definition of sensitivity:

$$\frac{\partial \Delta V^*}{\partial H_{AC}} = S_{AC} = S_0 + S_T \cdot H_{DC}$$

$$S_T = \frac{1}{H_{DC}}\left(\frac{\partial \Delta V^*}{\partial H_{AC}} - S_0\right)$$

$$S_T \partial H_{AC} = \frac{1}{H_{DC}} \partial \Delta V^* - \frac{S_0}{H_{DC}} \partial H_{AC} \tag{1}$$

and considering that S_T and S_0 are constants and integrating over H_{AC}:

$$S_T \int \partial H_{AC} = \frac{1}{H_{DC}} \int \partial \Delta V^* - \frac{S_0}{H_{DC}} \int \partial H_{AC}$$

$$\Delta V^* = S_T \cdot H_{DC} H_{AC} + S_0 H_{AC} \tag{2}$$

This definition proves to be more precise and practical as the theoretical response of the piezoinductive device relies on S_T and S_0, both of which are experimentally measurable parameters through sweeps in H_{AC} and H_{DC}.

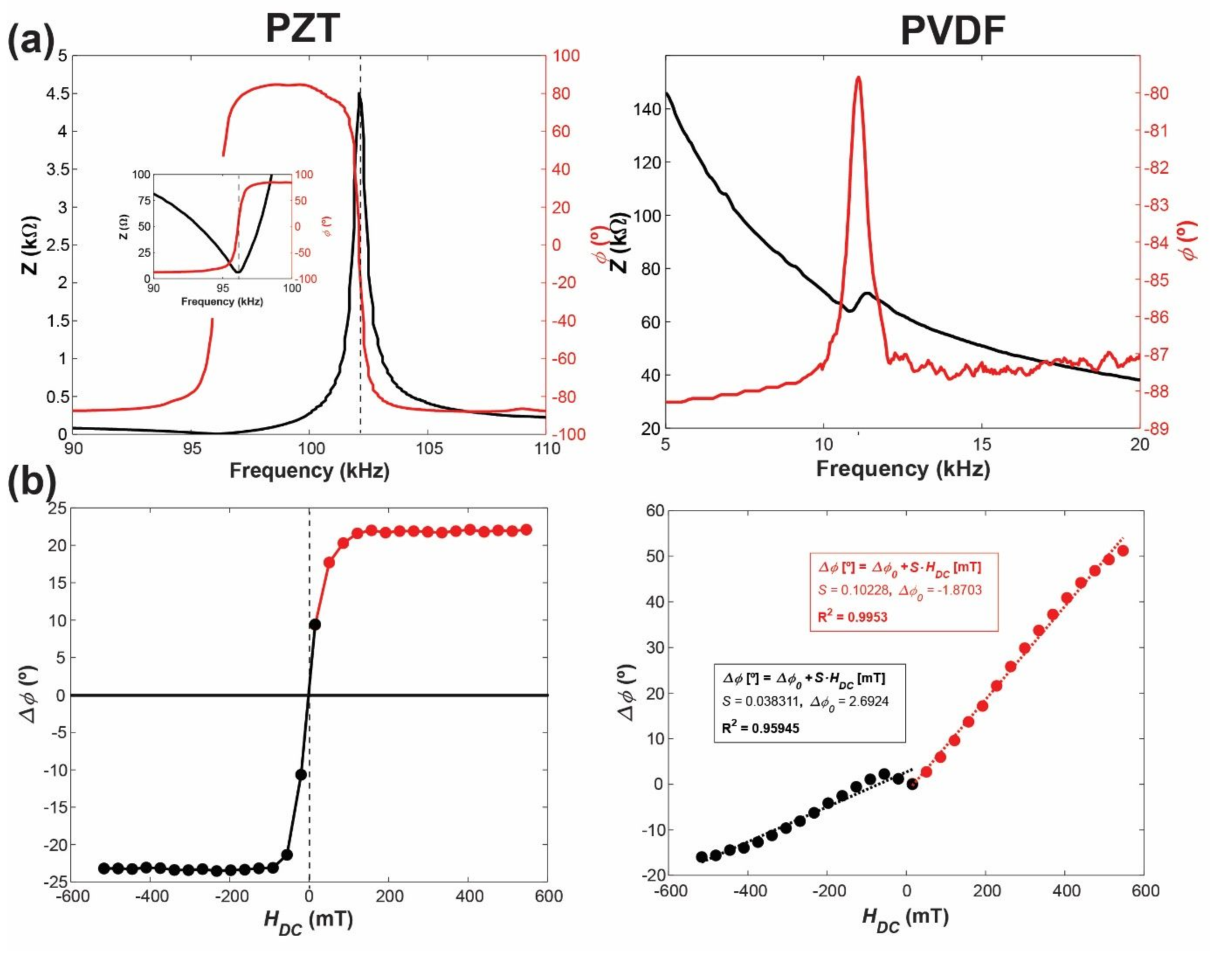

Figure S4. Comparison between a piezoelectric polymer PVDF sample and a ceramic PZT disc: a) impedance b) phase.
